# Outsourcing the Nucleus: Nuclear Pore Complex Genes are no Longer Encoded in Nucleomorph Genomes

**Published:** 2007-02-27

**Authors:** Nadja Neumann, Daniel C. Jeffares, Anthony M. Poole

**Affiliations:** 1 Department of Molecular Biology and Functional Genomics, Stockholm University, SE-106 91 Stockholm, Sweden; 2 Wellcome Trust Sanger Institute, Wellcome Trust Genome Campus, Hinxton, Cambridge, CB10 1SA, U.K

**Keywords:** nuclear pore complex, nucleomorph, nucleoporin, reductive evolution, gene loss

## Abstract

The nuclear pore complex (NPC) facilitates transport between nucleus and cytoplasm. The protein constituents of the NPC, termed nucleoporins (Nups), are conserved across a wide diversity of eukaryotes. In apparent exception to this, no nucleoporin genes have been identified in nucleomorph genomes. Nucleomorphs, nuclear remnants of once free-living eukaryotes, took up residence as secondary endosymbionts in cryptomonad and chlorarachniophyte algae. As these genomes are highly reduced, Nup genes may have been lost, or relocated to the host nucleus. However, Nup genes are often poorly conserved between species, so absence may be an artifact of low sequence similarity. We therefore constructed an evolutionary bioinformatic screen to establish whether the apparent absence of Nup genes in nucleomorph genomes is due to genuine absence or the inability of current methods to detect homologues. We searched green plant (*Arabidopsis* and rice), green alga (*Chlamydomonas reinhardtii*) and red alga (*Cyanidioschyzon merolae*) genomes, plus two nucleomorph genomes (*Bigelowiella natans* and *Guillardia theta*) with profile hidden Markov models (HMMs) from curated alignments of known vertebrate/yeast Nups. Since the plant, algal and nucleomorph genomes all belong to the kingdom Plantae, and are evolutionarily distant from the outgroup (vertebrate/yeast) training set, we use the plant and algal genomes as internal positive controls for the sensitivity of the searches in nucleomorph genomes. We find numerous Nup homologues in all plant and free-living algal species, but none in either nucleomorph genome. BLAST searches using identified plant and algal Nups also failed to detect nucleomorph homologues. We conclude that nucleomorph Nup genes have either been lost, being replaced by host Nup genes, or, that nucleomorph Nup genes have been transferred to the host nucleus twice independently; once in the evolution of the red algal nucleomorph and once in the green algal nucleomorph.

## Introduction

The nucleus is the defining feature of eukaryote cells, separating the genome from the cytoplasm, the site of protein synthesis. The nucleus is bounded by a double-membrane envelope studded with nuclear pore complexes (NPCs) that facilitate translocation of macromolecules between nucleus and cytoplasm. Studies from yeast and vertebrate model systems have built up a detailed picture of the NPC. The NPC contacts the membrane surface at the interface between the continuous inner and outer membranes and is anchored in place by a small number of transmembrane proteins, which are not universally conserved (Bapteste et al. 2005; Mans et al. 2004; Suntharalingam and Wente, 2003). It possesses 8-fold rotational symmetry (but is asymmetrical across the nuclear envelope) and is made up of 30–50 nucleoporin proteins (Nups), each present in multiple copies (Lim and Fahrenkrog, 2006; Suntharalingam and Wente, 2003). Recent bioinformatic screens have built on and extended experimental studies, identifying nucleoporin homologues across a wide range of eukaryotic taxa, and providing insight into the extensive conservation of this complex across the eukaryote domain (Bapteste et al. 2005; Mans et al. 2004). Despite overall conservation of the nuclear pore complex, many nucleoporins are poorly conserved at the sequence level, and basic BLAST-based screens do not readily recover nucleoporin genes from genomic data (Rose et al. 2004).

While identification of Nup homologues is not trivial, the consensus arising from the studies published to date is that numerous Nup genes are conserved across all eukaryotes. Possible exceptions to this are the nucleomorph genomes of the chlorarachniophyte alga *Bigelowiella natans*, and *Guillardia theta*, a cryptomonad alga. In contrast to other nuclear genomes thus far screened, nucleomorphs are remnant nuclei stemming from two separate secondary endosymbioses wherein a non-photosynthetic eukaryote cell engulfed a photosynthetic eukaryote (Archibald, 2005). In the case of *B. natans*, the nucleomorph is of green algal origin; the nucleomorph of *G. theta* is red algal in origin. In both cases, the nucleomorph genomes have undergone extreme reduction; the *G. theta* nucleomorph is a mere 551kb, compared to an estimated 350Mb for the host genome (Douglas et al. 2001), and a similar picture is seen for *B. natans*, where the nucleomorph genome is 373kb (Gilson et al. 2006).

Nucleomorph NPCs are potentially interesting for several reasons. If for some reason no Nup genes can be transferred to the main nucleus, genome reduction may have left nucleomorphs with only the most crucial components (a minimal NPC). Alternatively, it may be that only those genes that cannot be relocated to the main nucleus remain. This might be borne out if the pattern of Nup genes found in these two independent nucleomorph genomes is very similar. A second possibility is that there is no clear correspondence between which Nup genes are present; this may indicate that loss/relocation to the main nucleus is ongoing, the order of loss being largely stochastic. Third, there may be no detectable nucleomorph Nup genes in one or both genomes. This might indicate that all Nup genes have been relocated to the main nucleus, or that host Nup genes have replaced nucleomorph Nup genes with host Nups being imported into the endosymbiont.

Neither the genome annotations (Douglas et al. 2001; Gilson et al. 2006), nor a large scale PSI-BLAST analysis, which included *G. theta* (Mans et al. 2004), revealed evidence for nucleoporins in nucleomorphs. We therefore designed a bioinformatic screen to establish whether there were any nucleoporin genes in the *B. natans* and *G. theta* nucleomorph genomes. Importantly, we wished to be able to distinguish between absence of homologues from the genome and a failure to detect potential homologues owing to extensive sequence divergence (see screen design below).

We detect numerous Nup homologues in the genomes of *Cyanidioschyzon merolae* (red algae), *Chlamydomonas reinhardtii* (green algae), *Arabidopsis thaliana* and *Oryza sativa* (plants), but none in either nucleomorph. While we cannot formally exclude the possibility that Nup genes are present in the nucleomorph genomes and too divergent to detect, the design of our screen is such that this conclusion would necessitate a special case of extremely elevated evolutionary tempo to apply to two independently formed lineages, across multiple evolutionarily unrelated proteins. We therefore conclude that these genes have been completely lost or relocated to the main nucleus twice independently during evolution.

## Materials and Methods

### Screen design

**Figure 1:**
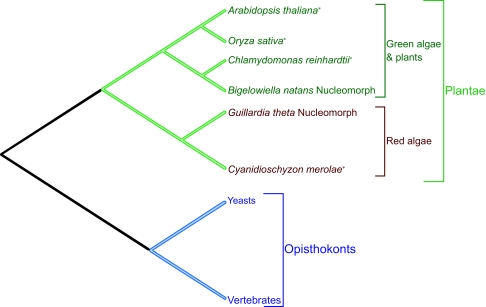
Screen design The tree shows phylogenetic relationships of the genomes used in this study, phylogeny from (Bhattacharya et al. 2004; Yoon et al. 2004). Note that the phylogeny indicates the evolutionary relationships between the red and green algal nucleomorphs and other members of the Plantae; the phylogenetic affinities of *B. natans and G. theta,* using genes from the main nucleus of these species, are not shown. The evolutionary distance from the known Opisthokont Nup genes to all study species within Plantae is expected to be comparable (see text). Profile HMM searches against *A. thaliana, O. sativa, C. reinhardtii and C. merolae* can therefore act as ‘positive controls’ to show that the searches are sufficiently sensitive to detect Nup genes in Plantae. For example, if the detection of Nups across the Opisthokont-Arabidopsis distance is achievable, it should likewise be possible to detect Nups across the Opisthokont-*Bigelowiella natans* nucleomorph distance. Profile HMM searches were thus carried out against those species marked with an asterisk (*), to establish that the search methods were sufficiently sensitive to span the evolutionary distance. The original profiles, and also profiles containing Nups identified from this screen were then used to search for Nups in both nucleomorph genomes. Candidate Nup sequences from the species indicated (*) were also used to search nucleomorph genomes by BLAST.

Given the possibility that nucleoporin genes may no longer reside in the nucleomorph genomes of *G. theta* and *B. natans*, we sought to construct a screen that could be interpreted in the event of a negative result (i.e. where no candidate Nup genes were detected in either nucleomorph genome). The logic is as follows, and is based on the known phylogenetic relationships of the plant, algal and nucleomorph genomes included in the screen (Bhattacharya et al. 2004; Yoon et al. 2004) (Figure 1).

In order to use maximal information, we used HMMER to generate profile hidden Markov models (profile HMMs) from a training dataset of known Nup proteins. All sequences in our training data are from the Opisthokonts (primarily yeast and vertebrates; see below), and both the target genomes (*G. theta* and *B. natans* nucleomorphs) are members of the Plantae (see Fig. 1a). Identification of nucleoporin genes in other members of the Plantae provides an internal positive control, showing that, for any given nucleoporin, genes in the Plantae can be identified using genes from Opisthokonts. Screening the genome sequences of the green plants *Arabidopsis thaliana, Oryza sativa* and the draft-assembly and the green alga *Chlamydomonas reinhardtii* thus serve as positive controls for detection of Nups in *B. natans* (green algae/green plant group), while the red alga *Cyanidioschyzon merolae* serves as a positive control for detection of Nup genes in *Guillardia theta* (red algae group).

The screen can thus be interpreted as follows. Strong candidates found in all control Plantae genomes should also be found in the nucleomorph genomes, if present. For those Nups where we do detect homologues in these genomes, but do not detect corresponding homologues in the nucleomorphs, we argue that this is indicative of genuine absence, rather than failure to detect homologues owing to insufficient sensitivity. In contrast, for those Nups where we identify no homologues in any of the Plant, free-living algal species, or the nucleomorphs, we cannot establish whether the result is due to genuine absence or an inability to detect homologues.

The screen can be further refined in two ways. First, candidate nucleoporin sequences from the plant or algal genomes can be added to each of the existing profiles, thereby including training data from the Plantae. The nucleomorph genomes can then be re-screened. As each sequence in a profile is given equal weighting under HMMER (see below), one may reason that the profiles are nevertheless not well trained for Plantae, though this seems unlikely if the other Plantae genomes can be successfully screened without iteration. This issue can be addressed by way of a BLAST screen that takes phylogenetic relationships into account; reciprocal BLASTs between plant and free-living algal genomes provides a means of demonstrating the capacity of BLAST to reciprocally recover candidates identified by HMMER. Direct screening with the most closely related sequences was thus carried out by way of BLAST-screens of red- and green-algal nucleomorph genomes using, respectively, plant, green- and red-algal nucleoporin candidates (Fig. 1b).

The design of this screen maximizes the chance of finding nucleoporin homologues in the two nucleomorph genomes, and furthermore, we believe this gives us sufficient information to directly interpret the absence of candidate nucleoporin orthologues from nucleomorph genomes as most probably genuine absence, not failure to detect homologues bioinformatically.

### Nup dataset

Vertebrate and yeast Nups previously identified through experiment (Suntharalingam and Wente, 2003) were recovered from Genbank. Additional vertebrate sequences were gathered by BLASTP against the NCBI nr database using the experimentally verified Nups as queries, and with strict criteria for acceptance (E-value ≤ 10^−100^ and bit score ≥ 200). Additional yeast sequences were recovered from the available yeast genome data (Cliften et al. 2003; Dujon et al. 2004; Kellis et al. 2003), collecting only yeast Nup genes that were in regions of conserved synteny with the orthologous *S. cerevisae* Nup gene as a further criterion to ensure that only orthologues were included in building the dataset.

Nup protein sequences were aligned with CLUSTALX (1.83) (Thompson et al. 1997), and manually vetted. As conservation between yeast and vertebrate proteins was in some cases poor, we created separate vertebrate and yeast alignments where necessary; for some Nups, conservation between vertebrates and yeast was sufficient to enable construction of reliable alignments across all species.

Profile HMMs were built from all alignments using HMMER 2.3.2 (http://hmmer.janelia.org). Both local and global profile HMMs were built for all alignments. Domain information for the different Nups was gathered from Swiss-Prot (http://www.expasy.org) (Boeckmann et al. 2003) and PFAM (http://www.sanger.ac.uk) (Finn et al. 2006), and from our own examination of all alignments.

### Genome screens

Protein-coding gene sets from *C. merolae* (Matsuzaki et al. 2004), *A. thaliana* (Arabidopsis-Genome-Initiative, 2000) and *C. reinhardtii* were searched with each profile using HMMSEARCH from the HMMER package. Results were evaluated according to E-value (accept ≤ 10^−10^) and reciprocal BLASTP searches against Genbank to establish whether new candidates successfully recovered experimentally-characterised Nups from the original alignments. Finally candidate Nups were aligned to known Nups using Clustal X to establish whether functional and evolutionarily-conserved domains were present. The *B. natans* and *G. theta* nucleomorph genomes were likewise screened in this way, accepting all hits regardless of E-value to produce an exhaustive search.

Rice Nup candidates were identified with HMMSEARCH search against the TIGR rice annotation, release 3.0 (Yuan et al. 2005), and from the KOME rice cDNA database (Kikuchi et al. 2003) using TBLASTN (Altschul et al. 1997) with each *Arabidopsis* protein as query, accepting only hits with E value ≤ 10^−20^. Reciprocal BLASTX searches were carried out with all rice cDNA candidates against the *Arabidopsis* genome to exclude potential false positives. Rice candidate protein sequences were recovered from NCBI and verified by pairwise alignment to each *Arabidopsis* candidate.

Candidates from *A. thaliana*, rice and *C. merolae* were added to the alignments and new profiles were created. The two nucleomorph genomes (*G. theta* and *B. natans* (Douglas et al. 2001; Gilson et al. 2006)) were re-screened with HMMSEARCH using these new yeast-vertebrate-Plantae profiles as described above. In addition, Nup candidates from *A. thaliana*, rice, *C. reinhardtii* and *C. merolae* were used in BLAST searches of the nucleomorph genomes, using a range of substitution matrices (data not shown). Choice of matrix did not affect results. Alignments and profiles are available on request.

Genome/protein coding sequences were obtained from the following sources: *Cyanidioschyzon merolae* (Matsuzaki et al. 2004) (http://merolae.biol.s.u-tokyo.ac.jp/-version 17 September, 2005), *Arabidopsis thaliana* (Arabidopsis-Genome-Initiative, 2000) (ftp://ftpmips.gsf.de-version 2 February, 2004), *Oryza sativa* L. ssp. japonica (International-Rice-Genome-Sequencing-Project, 2005; Yuan et al. 2005) (http://www.tigr.org-version 3.0), *Chlamydomonas reinhardtii* **(**http://genome.jgi-psf.org/-version 3.0**)** ; *Guillardia theta* (Douglas et al. 2001) (ftp://ftp.ebi.ac.uk-version 4 January, 2005); *Bigelowiella natans* (Gilson et al. 2006). (Genbank acc. nos. DQ158856, DQ158857, DQ158858).

### Verification of candidate Nups

Nup candidates were searched against the Genbank nr-database with BLAST to establish whether the most similar proteins in the database were Nups. Candidates were also incorporated into CLUSTALX alignments and examined for conserved regions. BLAST and BLAST2SEQ (http://www.ncbi.nlm.nih.gov/BLAST) were used in some cases to aid in checking that domains were present and correctly aligned.

## Results and Discussion

**Figure 2:**
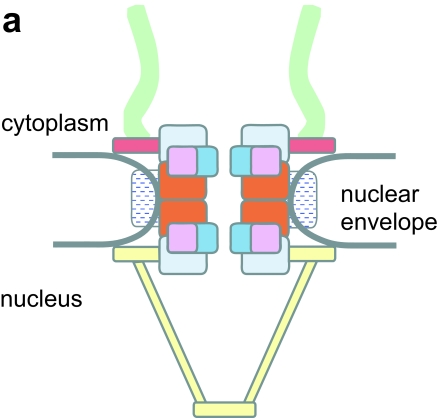

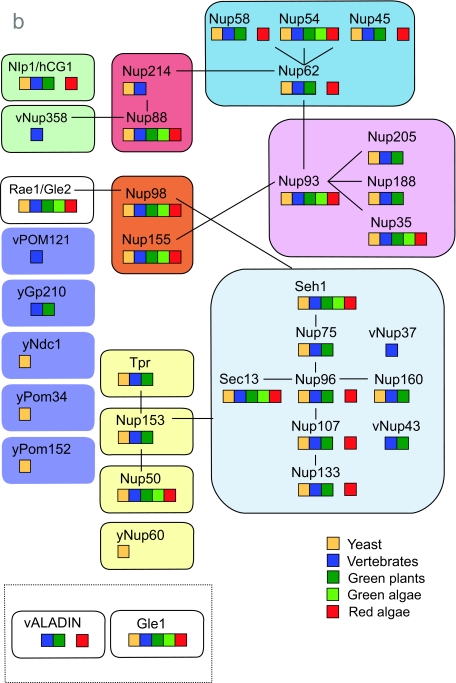
Position of identified plant, green- and red-algal Nups in the NPC **a.** Schematic of the nuclear pore complex. Colour scheme shows the main features or protein complexes (illustrated in b). **b.** The majority of nucleoporin genes are conserved between Opisthokonts and Plantae. Nucleoporins are grouped according to known mammalian protein subcomplexes (coloured rounded boxes). Location of subcomplexes in the mammalian nuclear pore complex is indicated by colour-coding in a (above). Coloured squares within the rounded boxes indicate the phylogenetic distribution of each Nup gene, as established in this study (see Table 1). White boxes indicate that either the position of these Nups in the NPC is not known, that they are dynamic, or that they appear in different locations between yeast and vertebrates. Black lines indicate known biochemical interactions in the vertebrate NPC (for clarity, known interactions between yeast proteins are not shown). Proteins with prefix y have been identified in yeast but not vertebrates. Proteins prefixed with v are found in vertebrates but not yeast. All other proteins are found in both groups. Nup45 and 58 are generated by alternative splicing and are coded by the same gene (Hu and Gerace, 1998). We screened using Nup58/Nup49 sequences since Nup45 is identical to Nup58 save for a truncated C-terminal region. Our screen cannot establish the existence of splice variants; we thus count Nup58 and Nup45 as a single candidate in our screens and include Nup45 only for completeness as the figure is based on (Lim and Fahrenkrog, 2006). Additional information sourced from (Suntharalingam and Wente, 2003).

Our HMMER screen using Opisthokont Nup protein sequences successfully detected green plant candidates for all nucleoporins conserved between vertebrates and yeasts, with the exception of Nup214 (Table 1 and Fig. 2). Broadly speaking, we observe that the central regions of the NPC are the most universally conserved amongst taxa and produced the highest bit scores from hmm searches. No yeast-specific nucleoporin genes were identified among the Plantae, but 3 of 6 vertebrate-specific Nup genes (Gp210, Aladin and Nup43) were found in green plants with one of these, Aladin, also present in *C. merolae*. From the preliminary data available from the *C. reinhardtii* genome project, we identified 11 candidate nucleoporin genes, whereas 18 candidates were identified in the complete genome of *C. merolae*. The results we report for *A. thaliana*, *O. sativa* and *C. merolae* broadly agree with the psi-BLAST survey reported by Mans et al. (Mans et al. 2004). Significantly, we find candidates for five Nups not identified by these authors: Nup2/50, Nup42/Nlp1, Nup 45/58/49 in *Arabidopsis*, rice and *C. merolae*; Nup 1/153 in *Arabidopsis* and rice and Nup133 in *C. merolae* (Table 1).

In contrast to the essentially complete complement of candidate green plant Nups, we find a more limited set in the red and green algae. In both cases, we screened available predicted protein-coding genes. However, since the *C. reinhardtii* data are preliminary, this may account for the lower number of candidates in this species. Given that there are several cases where we detect green plant and red algal Nup homologues, with no corresponding candidate in *C. reinhardtii* (Aladin, Nup62, Nup 107, Nup133), this may be due to incomplete, or absent gene predictions. Indeed, we find no examples of Nup genes conserved in green plants and *C. reinhardtii* to the exclusion of the red alga. BLASTing the two algal genomes with Nup candidates from plants did not increase the number of candidates, leading us to conclude that, at least for *C. merolae*, we have a fairly complete set, with all the major sub-complexes represented (Fig. 2). Whether the remainder are simply too divergent at the sequence level to be detected cannot be ascertained.

This analysis identifies 18 Nups conserved between Opisthokonts, green plants and the red algal lineage, of which 11 are also found in the green alga. For plants, we find 26 Nup homologues. Our screen for Nup candidates in the Plantae is thus the most comprehensive to date. These data can now be brought to bear on our nucleomorph genome screen.

**Figure 3:**
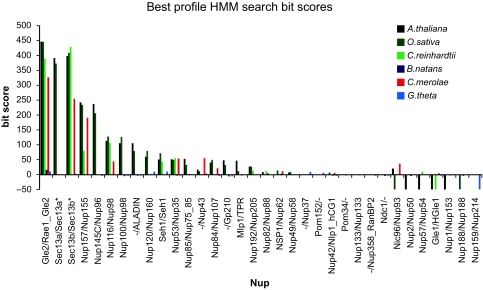
Nup bit scores from HMM searches HMM searches against the nucleomorph genomes (*Bigelowiella natans* and *Guillardia theta*) did not detect any Nup candidates even for Nups that were clearly identified in all examined Plantae genomes. The plot shows the single best bit score amongst the yeast/vertebrate local/global searches for each species. Bit score values <–50 are not shown to scale (see supplementary table 1).

Profile hmm searches detected no strong Nup candidates in either the *B. natans* or the *G. theta* nucleomorph genomes (Fig. 3). This is the case even for the 11 Nup profiles that returned homologues in all four control genomes (*Arabidopsis*, rice, *C. merolae* and *C. reinhardtii*). Neither did adding all candidate Plantae Nup orthologues to our profile HMMs and re-screening alter this result.

To determine whether Nup genes were present but highly divergent from vertebrate/yeast Nup genes, we examined all hits returned from the nucleomorph genome searches for conserved sequence stretches regardless of E-value/bit score (see Supplementary Table 1). We did this by pairwise local alignment, and by global alignment (CLUSTALX) with all sequences for a given Nup orthologue. The top nucleomorph candidates for each nucleoporin were also BLASTed against the Genbank nr database to establish whether any similar proteins were annotated as nucleoporins, or whether candidates appeared more similar to other known proteins. This exhaustive examination of possible Nup candidates failed to identify any nucleoporin genes in either of the nucleomorph genomes. The majority of the weak candidates could be clearly identified as proteins other than Nups by BLASTs against the Genbank nr database (Supplementary Table 1).

The lack of detectable Nup genes in nucleomorph genomes is in stark contrast to the clear conservation of numerous nucleoporins between green algal/plant, red algal and opisthokont genomes. Given the phylogenetic relationships of the species involved, and the successful detection of 11 Nup genes in all four positive control genomes (Fig. 2b), we conclude that no genes in either nucleomorph code for Nups.

The absence of nuclear pore genes in nucleomorph genomes suggests three possible scenarios: fast evolution of nucleomorph Nup genes, transfer of Nup genes to the host nucleus, or loss and concurrent replacement of nucleomorph Nup genes by those encoded by the host nucleus. The possibility that the NPC in nucleomorphs is composed of non-homologous proteins, having evolved twice, is not a likely scenario, given the presence of identifiable Nup homologues across much larger evolutionary distances on the eukaryote tree (Bapteste et al. 2005; Mans et al. 2004). Our own analyses of other eukaryote genomes using the profile HMMs generated for this study are consistent with these earlier results (data not shown).

The first scenario, that nucleomorph sequences have evolved so rapidly that they are undetectable by our methods, is plausible in principle, but unlikely. Nucleomorph genomes are known to be fast evolving, probably due to their asexual life style, which renders them prone to Muller’s ratchet (Gilson and McFadden, 2002). Analysis of nucleomorph genes for proteins that function in the periplastid space for which nuclear homologues also exist (Patron et al. 2006), showed that the average pairwise distance was higher between the *B. natans* nucleomorph and both *A. thaliana* and *C. reinhardtii* than between nuclear homologues of the latter two genomes, with this trend being more pronounced than for comparisons between the *G. theta* nucleomorph and *C. merolae* and *A. thaliana*.

However, invoking an elevated mutation rate to account for the apparent absence of Nups in the nucleomorph genomes (i.e. that Nups are present but not detectable) would require two conditions not supported by current data. First, the rate of sequence evolution would have to be sufficiently high that sequence similarity degraded to undetectable levels since the evolutionary split between the ancestors of the *B. natans* nucleomorph, the plants and *C. reinhardtii*, likewise for the split between the *G. theta* nucleomorph and *C. merolae*. Second, amino acid sequence conservation in *all* nucleoporin proteins would need to be sufficiently unimportant for function that it would be possible for a given Nup to become so divergent in sequence as to be unrecognisable. This appears not to be the case for the majority of Nups, which are detectable across all eukaryote kingdoms where genome sequence is available (Mans et al. 2004) and despite clear differences in rates of sequence change between unrelated Nups (Bapteste et al. 2005).

**Figure 4:**
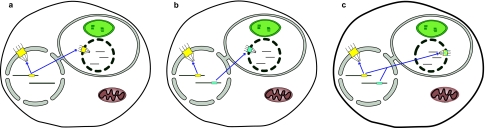
Scenarios describing the possible makeup of nucleomorph NPCs As no genes for nucleoporins were detected in the nucleomorph genomes of either Bigelowiella natans or Guillardia theta, the expectation is that the genes for the nucleomorph NPC are in the main nucleus. The figure illustrates three possible scenarios. **a.** Nucleomorph NPC genes have been completely lost. In this scenario, the nuclear pore complexes of the main nucleus and the nucleomorph are identical, with one set of genes (those which are of host origin) coding for both NPCs. **b.** Nucleomorph NPC genes have been relocated to the main nucleus. The NPCs of the main nucleus and the nucleomorph are thus distinct, and belie their separate evolutionary origins. **c.** Nucleomorph NPCs are to some degree chimaeric. Under this model, a combination of NPC genes of nucleomorph and host origin would contribute to the make up of the nucleomorph NPC.

The fact that we see such a clear contrast between our capacity to detect Nups in all available genomes from the Plantae but not in the two nucleomorph genomes leads us therefore to conclude that Nup genes are no longer residing in either of nucleomorph genomes. Based on the other examples of gene relocation to the host nucleus in primary endosymbioses, we expect nucleomorph Nup genes have either been relocated to the main nucleus with these proteins being imported into the nucleomorph, or they have been lost altogether, with those encoded in the host genome functioning in both the main nucleus and the nucleomorph (Fig. 4).

‘Sharing’ of Nup genes between host and nucleomorph would require dual targeting of nucleoporins to the nucleomorph and to the main nuclear envelope after translation in the cytoplasm of the host. Dual targeting of nuclear encoded proteins to both mitochondria and chloroplasts is known in plants (Mackenzie, 2005), so is not without precedent. Import of proteins into the chloroplast in *B. natans* requires bipartite leader sequences composed of a signal peptide (for trafficking through the endoplasmic reticulum) and a transit peptide (for entry into the chloroplast) (Rogers et al. 2004). This implies that import of proteins destined for the nucleomorph, should only require a signal peptide. Surprisingly, this is not the case in *G. theta*, where it has recently been demonstrated that nucleus-encoded sequences targeted to the periplastid compartment are in fact bipartite; whether sequences are targeted to the periplastid compartment or to the plastid can be attributed to a single amino acid in the bipartite leader sequences (Gould et al. 2006). In principle, alternative splicing could enable differential targeting in either species.

One final possibility is that nucleomorph NPCs are chimaeric in nature; that is, some nuclear-encoded nucleoporins are of nucleomorph origin, and some are of host cell origin, being targeted to both the nucleomorph and main nucleus. Overall, it is expected that large multisubunit complexes are refractory to chimaerisation because of coevolution of interacting proteins (Jain et al. 1999). Mammalian mitochondrial ribosomal proteins, which, being nucleus-encoded and part of a large multisubunit complex, provide a close analogy to the NPC and fit the scenario given in figure 4b. However, it is also evident that a significant number of proteins with no homology to either bacterial or eukaryotic ribosomes have been recruited into mammalian mitochondrial ribosomes (O’Brien, 2002), demonstrating that these structures are far from static. Moreover, single instances of chimaerism have been observed in plant mitochondrial ribosomes. A duplicated homologue of ribosomal protein S13 of chloroplast origin has been recruited into the mitochondrial ribosome in *A. thaliana* (Adams et al. 2002; Mollier et al. 2002). Duplication of cytosolic S8 has likewise led to replacement of the mitochondrial counterpart, again in *Arabidopsis* (Adams et al. 2002). We therefore think it is premature to rule out some degree of chimaerism (Fig. 4c), and note that this would be more likely at the periphery, for instance at the cytoplasmic fibrils, where the extent of sequence conservation and species distribution among eukaryotes is low (unpublished observations).

Establishing whether nucleoporins of nucleomorph origin have been completely lost, or whether there are two sets of genes for nucleoporins, one for each nuclear compartment, and whether red- and green-algal nucleomorphs differ in this respect must await the sequencing of the main nuclear genomes from *G. theta* and *B. natans.*

To conclude, our analysis indicates that convincing evidence for the absence of genes can be distinguished from a difficulty in detecting genes due to low sequence similarity. The use of sister taxa as ‘positive controls’ for genome searches, combined with the sensitivity of hmm searches provides a conclusive method to characterize gene loss in completed genomes. We used this method to show probable loss of all nucleoporin genes independently from green and red algae nucleomorphs. The loss of these genes from nucleomorphs is consistent with the strong pressure for genome minimization in these genomes (Douglas et al. 2001; Gilson and McFadden, 2002; Gilson et al. 2006), and indicates that eukaryote genome miniaturization may proceed with some recurrent events.

## Figures and Tables

**Table 1:** Nucleoporin genes in identified in screened Plantae genomes Nups identified using profile HMM and BLAST searches in different Plantae species. Our screen identifies a set of 26 nucleoporins in Arabidopsis and rice, 11 in C. reinhardtii and 18 in C. merolae. Genbank gi/Accession numbers for all candidates are included where available.

NUPS		*A. thaliana*^a^	*O. sativa*^b^	*C. reinhardtii*^c^	*C. merolae*^d^
Vertebrate	Yeast				
Nup153^e^	Nup1	At3g10650	AAT75265	––	––
		gi:37202004	gi:50355740		
Nup62	NSP1	At2g45000	AK064176^f^	––	CMP228C
		gi:30689895	gi:32974194		
Nlp1/hCG1	Nup42	AT1G75340	BAD54049	––	CMG145C
		gi:10120449	gi:53791927		
Nup35	Nup53, 59	At3g16310	BAD73206	Chlre2_kg.scaffold_20000237	CML289C
		gi:18401087	gi:56202114		
Nup54	Nup57	At1g24310	XP_506270	fgenesh2_pg.C_scaffold_20000065	CMM220C
		gi:15221725	gi:51963372		
Nup88	Nup82	At5g05680	XP_473828	estExt_fgenesh2_pg.C_260018	CMO203C
		gi:15239202	gi:50928601		
Nup107	Nup84	At3g14120	ABA95041	––	CMC129C
		gi:15231787	gi:77552244		
Nup75/85	Nup85	At4g32905	NP_916074	––	––
		gi:18418112	gi:34909454		
Nup93^g^	Nic96	At2g41620	Ak099999	fgenesh2_pg.C_scaffold_96000002	CMR125C
		gi:18405761	gi:32985208		
		At3g57350			
		gi:15230280			
Nup96^h^	Nup145	At1g80680	ABF94225	––	––
		gi:30699531	gi:108706430		
Nup98^i^	Nup100, Nup116	At1g59660	BAD68826	gwW.7.237.1^j^	CMB112C
		gi:15218866	gi:55297169		
Nup98^i^	Nup100, Nup116	At1g10390	ABA95896	––	––
		gi:22329468	gi:77553100		
Nup160	Nup120	At1g33410	XP_464115	––	––
		gi:15217540	gi:50905253		
Nup133^k^	Nup133	At2g05120	AAN52748	––	CMQ238C
		gi:15224474	gi:24308625		
Nup155	Nup157/170	At1g14850	BAD_62392	estExt_fgenesh2_pg.C_220139	CMH179C
		gi:5223918	gi:54290722		
Nup214/CAN	Nup159	––	––	––	––
Nup188	Nup188	At4g38760	NP_919901	––	––
		gi:15234783	37531198		
Nup205	Nup192	At5g51200	AK071672	––	––
		gi:30696017	gi:32981695		
HGle1	Gle1	At1g13120	XP_466532	Chlre2_kg.scaffold_12000184	CMS459C
		gi:15222184	gi:50910087		
Rae1/Gle2	Gle2	At1g80670	XP_480345	estExt_gwp_1W.C_380004	CMI077C
		gi:15220198	gi:50941635		
TPR	Mlp1	At1g79280	XP_467721	––	––
		gi:15219336	gi:50912627		
Seh1^l^	Seh1	At1g64350	BAD61535	fgenesh2_pg.C_scaffold_19000005	––
		gi:18408028	gi:54290874		
sec13^l^		At2g30050	XP_506712	estExt_gwp_1W.C_540024	CMJ112
		gi:15227692	gi:51963858		
		At3g01340	XP_477253		
		gi:15232095	gi:34897242		
	Nup60	––	––	––	––
	Ndc1	––	––	––	––
	Pom34	––	––	––	––
	Pom152	––	––	––	––
Pom121		––	––	––	––
Gp210		At5g40480	BAD46654	––	––
		gi:15242716	gi:52076141		
					
Nup358/		––	––	––	––
RanBP2					
ALADIN		At3g56900	ABA91372	––	CMM309
		gi:15230151	gi:77548575		
Nup37		––	––	––	––
Nup43		At4g30840	XP_481137	––	––
		gi:18417678	gi:50943219		
Nup50^m^	Nup2	At4g11790	XP_469196	––	CMH178C
		gi:18413658	gi:50917599		
Nup58/ Nup45^n^	Nup49	At4g37130	BAD38027	––	CMS092C
		gi:15235442	gi:51535945		

a Protein nomenclature taken from genome annotation ftp://ftpmips.gsf.de download version arabi_all_proteins_v020204.

b Protein nomenclature taken from *O. sativa* genome annotation at http://www.tigr.org, version 3.0.

c Protein nomenclature taken from genome annotation http://genome.jgi-psf.org download version *C. reinhardtii* v3.0

d Protein nomenclature taken from genome annotation from http://merolae.biol.s.u-tokyo.ac.jp/

e Candidates for Nup1/Nup153 in plants are poorly conserved.

f None of the sequences in NCBI corresponded exactly to the sequence obtained from the rice genome, the gi number corresponds to the best hit using blast.

g We identified two candidates to Nup93 in *A. thaliana*. These are identical to the two Nup93 candidates reported by (Mans et al. 2004).

h BLAST2SEQ alignments with the human Nup98–96 precursor (gi:33860189) suggest that our sequences correspond to the C-terminal part of the precursor that encodes Nup96. They contain furthermore the nucleoporin autopeptidase domain in their N-terminal end.

i BLAST2SEQ alignments with the human Nup98–96 precursor (gi:33860189) indicate that our sequences correspond to the N-terminal part of the precursor that encodes Nup98.

j Probably partial sequence only. It corresponds to the autopeptidase domain of the nup98–96 precursor.

k Candidate for Nup133 in *C. merolae* is poorly conserved.

l Seh1 and Sec13 are very similar in sequence, consequently it is difficult to establish orthology from sequence alone. Candidates are grouped according to greater similarity to either the sec13 (gi:544501) or the seh1 (gi:1322639) protein sequence of *S. cerevisiae* using BLAST2SEQ. Sequences might nevertheless be cryptic paralogues.

m CMH178 is a weak candidate. It possesses a domain homologous to the Ranbp1 domain in Nup50/2. However, outside this domain similarity to known Nup50/2 sequences is low.

n FG repeats regions are difficult to align across kingdoms and are present only in the C-terminus in *Arabidopsis* and rice candidates. CMS092C is a weak candidate as it does not contain recognisable FG repeats, in contrast to the green plant candidates.
